# Isolation of a new HIV-2 group in the US

**DOI:** 10.1186/1742-4690-5-103

**Published:** 2008-11-14

**Authors:** Stephen M Smith, Deanna Christian, Valéry de Lame, Urvi Shah, Louise Austin, Rajeev Gautam, Aarti Gautam, Cristian Apetrei, Preston A Marx

**Affiliations:** 1 Division of Infectious Diseases, Saint Michael's Medical Center, Newark, New Jersey, 07102, USA; 2 Divisions of Comparative Pathology and Microbiology, Tulane National Primate Research Center, Covington, LA 70433, USA; 3 Department of Tropical Medicine, School of Public Health, Tulane University, New Orleans, LA 70112, USA

## Abstract

Human immunodeficiency virus type 2 (HIV-2) emerged following cross-species transmission of simian immunodeficiency virus (SIV) from sooty mangabeys to humans several decades ago. The epidemic groups of HIV-2 have been established in the human population for at least 50 years. However, it is likely that new divergent SIVs can infect humans and lead to new outbreaks. We report the isolation of a new strain of HIV-2, HIV2-NWK08F, from an immunodeficient Sierra Leone immigrant. Health care providers in Sierra Leone and elsewhere need to be alerted that a subtype of HIV-2, which is not detected by PCR for epidemic HIV-2 strains, exists and can lead to immunosuppression.

## Correspondence

Infection with human immunodeficiency virus type 2 (HIV-2) is endemic in some countries of West Africa. Unlike infection with HIV type 1 (HIV-1), this infection has not appreciably spread beyond this area. The incidence of HIV-2 infection has even declined over the last 16–20 years [1,2]. The majority of human infections are caused by groups A or B, which have been referred to as the epidemic groups. The rate of progression to acquired immunodeficiency syndrome (AIDS) for the epidemic strains is not well defined[3]. However, variation in envelope during infection is similar to that seen in HIV-1[4]. Infections with non-epidemic subtypes (C-G) are known only as single person infections and available evidence indicates that infection did not lead to immune suppression[5]. The one noted exception is a group H virus, which caused immunodeficiency in a man from the Ivory Coast[6].

Sixteen years ago, infection with HIV-2 Group F was described in one individual from the northern province of Sierra Leone[7]. HIV-2 Group E was also found in a single person originating from Sierra Leone and was reported 18 years ago[5]. Virus was not isolated from either person, despite repeated attempts. Both individuals were healthy during the time of observation. Here we present evidence that a Group F virus isolated in 2008 appears to be a newly emerging HIV-2 group. The virus, HIV-2-NWK-08F, was isolated from a man with CD4 T-cell lymphopenia.

Patient X is a 68 year old male from Freetown, Sierra Leone. Patient X immigrated to the New Jersey, USA. in 2007. During the immigration process, he tested positive for antibodies against HIV. He was referred to the Peter Ho Memorial Clinic in Newark, New Jersey for follow-up and treatment in early 2008. Patient X's serum was repeatedly reactive by serological testing with ELISA kits containing HIV-1 and HIV-2 antigens. The western blot for HIV-1 was negative. His HIV-1 viral load was <48 copies and polymerase chain reaction (PCR) for HIV-1 proviral DNA was negative. An HIV-2 immunoblot was positive. The presumptive diagnosis was that Patient X had an HIV-2 infection. However, a PCR assay from a commercial laboratory for HIV-2 proviral DNA was negative (LabCorp, Research Triangle Park, NC). This result suggested one of two possibilities:

1. The proviral load was below the limit of detection of the assay.

2. The virus was too divergent from known HIV-2 epidemic groups to be amplified by the gag primers based on epidemic subtype consensus sequence.

**Figure 1:**
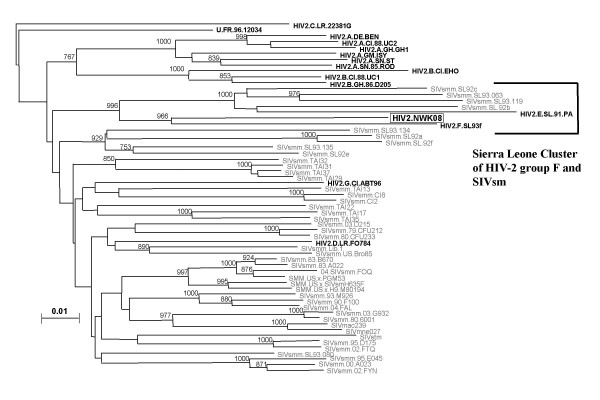
***gag *****phylogenetic tree showing highly significant branching order for a Sierra Leone group of SIV and HIV-2.** The Sierra Leone group includes 4 sooty mangabey SIVs and 2 other HIV-2s.

Patient X had a CD4 T-cell count of 338 cells/μl and a CD4:CD8 ratio of 0.52. This CD4 T-cell lymphopenia suggested that Patient X was actively infected with a divergent strain of HIV-2. To determine if Patient X had active infection with a non-epidemic strain, we attempted to isolate the virus and performed PCR with primers that were used in our previous study of HIV-2 in Sierra Leone[7]. On four separate occasions, we co-cultured Patient X's peripheral blood mononuclear cells (PBMC) with either PHA-stimulated normal donor PBMC (three different donors) or CEM-x-174 cells. Each culture resulted in virus production as measured by simian immunodeficiency virus (SIV) p27 gag EIA (Zeptometrix, Buffalo, NY). Using PCR we amplified *env *and *gag *of this provirus with subtype F primers. To rule out the possibility of PCR contamination, the *env *region was independently amplified in two laboratories, one in Newark, New Jersey and the other in Covington, Louisiana. The *env *sequence data were identical. Figure 1 shows the results of a phylogenetic analysis of *gag*. HIV-2NWK08F clusters significantly with six other viruses, all from Sierra Leone. Two viruses were found in household pet sooty mangabeys which are native to the region. A third was HIV-2 subtype E; a fourth was subtype F, from a woman who lived in the northern province of Sierra Leone – the same area as the original home of patient X.

Subtype F HIV-2 has not previously been known to cause immune suppression nor has it been known to be transmitted from person to person. It is not known how patient X acquired HIV-2-NWK-08F. Patient X denied exposure to monkeys. He denied ever hunting game. He had no tattoos, no history of needle exposure in Sierra Leone and no history of blood or blood product transfusion. Patient X reported only one sexual contact, his wife. No relative was available for testing. HIV-2-NWK-08F clusters most closely with HIV-2SL93F and next most closely with the 2 SIVs found in sooty mangabeys in Sierra Leone (Figure 1). A real time PCR protocol to quantify provirus was developed with *env *primers and probe. Patient X had a proviral load equal to 6,100 copies per 10^6 ^PBMC.

It is alarming that Patient X's virus was easily isolated and that his CD4 T-cell count is decreased with an abnormal CD4:CD8 ratio. Patient X's reported lack of exposure to pet monkeys or by hunting is also a concern, since it implies human to human transmission. Two recent studies of HIV-2 infected individuals found the median proviral load to be ~300 copies per 10^6 ^PBMC[8,9]. The proviral load in Patient X was significantly higher, indicating that this virus may have greater pathogenicity than most HIV-2 isolates. Together, these data suggest that HIV2-NWK08F is pathogenic and spreading within the human population. Previous infections with highly divergent strains have been thought to occur after transmission from monkey to human and represented "dead-end' infections, resulting in neither disease nor horizontal transmission.

Furthermore, the commercial assay for establishing the existence of active infection, namely PCR for HIV-2 proviral DNA, did not detect the provirus of this isolate. This result, similar to problems with early viral load assays measuring non-subtype B HIV-1 viremia[10], indicates that persons infected with this divergent HIV-2 group F will not be accurately diagnosed. A falsely negative PCR result may lead clinicians to infer that an individual's infection is latent or that the antibody tests are false positives.

These data demonstrate that a pathogenic, novel strain of HIV-2 is circulating, at least, within Sierra Leone. Health care providers in Sierra Leone and elsewhere need to be alerted that a strain of HIV-2, which is not detected by PCR for epidemic HIV-2 strains, exists and can lead to immunosuppression. Epidemiologic studies are required to determine the extent of this virus' spread in Sierra Leone and to other countries.

## Consent

Verbal consent was obtained from this patient by SMS. The consent was witnessed by VDL. The consent is available for review by the Editor-in-Chief of Retrovirology.

## Competing interests

The authors declare that they have no competing interests.

## Authors' contributions

SMS conceived of the study, designed most of the experiments and wrote the manuscript. DE isolated the virus. VDL developed the real-time PCR protocol. US assisted with cloning *env*. LA recognized the possibility that the patient was HIV-2 infected and provided valuable demographic data. RG, AG, CA, and PAM amplified *gag *and performed the phylogenic analysis. All authors read and approved the final manuscript.
